# The Effect of Interventions Led by Community Pharmacists in Primary Care for Adults with Type 2 Diabetes Mellitus on Therapeutic Adherence and HbA1c Levels: A Systematic Review

**DOI:** 10.3390/ijerph19106188

**Published:** 2022-05-19

**Authors:** Sarah Al Assaf, Romana Zelko, Balazs Hanko

**Affiliations:** University Pharmacy Department of Pharmacy Administration, Semmelweis University, 1092 Budapest, Hungary; sarah.assaf@phd.semmelweis.hu (S.A.A.); hanko.balazs@pharma.semmelweis-univ.hu (B.H.)

**Keywords:** therapeutic adherence, type 2 diabetes mellitus, pharmacist, strategies, systematic review

## Abstract

Type 2 diabetes mellitus has been assessed as a widespread disease globally. Unfortunately, this illness can occasionally go undetected and without symptoms until it reaches the emergency condition, and this can be notably true in patients who do not receive routine medical care. Pharmacists are the foremost accessible health care providers. They can help patients select the most appropriate hypoglycemic management strategy through their experiences. This review aimed to provide an overview of the literature published on community pharmacists’ interventions that are currently used and their usefulness in improving patient adherence and glycosylated hemoglobin (HbA1c) levels. Relevant studies were retrieved through a comprehensive search of three databases, PubMed/Medline, Web of Science, and CINAHL (2010 to 2020). In total, 8362 publications were identified. The final protocol was based on the “Preferred Reporting Items for Systematic Review and Meta-Analysis (PRISMA)”. After applying inclusion and exclusion measures, 21 articles were deemed relevant. In pharmacists’ interventions in diabetes care, patient education and counseling were the most common intervention methods. Essentially, this systematic review provides evidence and identifies the key features that may predict success in enhancing clinical outcomes and patient adherence to treatment. Based on our findings, we suggest further investigations of the root causes of non-adherence problems.

## 1. Introduction

Diabetes is a lifelong condition, and its proper management requires the individual’s active participation through the performance of self-care behaviors, such as adherence to therapy, exercise, diet, self-monitoring of blood glucose (SMBG), and foot care [1]. Type 2 diabetes mellitus is the utmost popular form of diabetes mellitus (DM), which represents 90–95% of totally diabetic patients [2]. Indeed, it is reaching epidemic proportions, as its prevalence increases at an alarming rate in developed and developing countries [3].

Recent appraisals specify that in 2021, there were around 537 million people that have diabetes, and this number is predicted to upsurge to 643 million by the year 2030 [4]. For this reason, public health has considered that type 2 diabetes mellitus disease is one of the furthermost remarkable emerging issues, which needs to be focused on and requires immediate attention.

The adherence to therapies is a primary core of treatment achievement that is defined according to the WHO as “the degree to which the persons’ behavior corresponds with the agreed recommendations from the health care providers” [5].

Unfortunately, 30 to 50% of chronic disease prescriptions have not been administered as recommended in developed countries [6]. Therefore, failure to therapeutic adherence could become a severe problem for patients with chronic diseases (e.g., diabetes, osteoporosis, heart disease, cancer, and asthma).

Several causes lay behind this. The results underline the importance of pharmacists’ intervention in type 2 diabetic patients’ therapeutic outcomes. Therefore, the authors propose the implementation of this into the regular pharmacists’ services on a regulatory basis, guided by professional protocols. The most significant non-adherence problem involves taking complex regimes for a long time. In this case, the patient will not realize the accurate dose and time of taking medications as the physicians or pharmacists recommended it [7,8].

The treatment approach for type 2 diabetes mellitus includes several conventional therapies, namely miglitol and acarbose, which delay the absorption of carbohydrates from food intake, sulfonylureas, which enhance insulin secretion, troglitazone, which increases insulin action in fat and muscle and metformin, which enhances the insulin mechanism in liver tissues [9,10,11,12].

The medication used to treat type 2 diabetes mellitus has limitations in the sense that it has side effects, especially when the patients have other medical conditions.

For example, episodes of hypoglycemia are slightly more common when using sulfonylureas than when using metformin. In addition, pioglitazone may slightly increase the risk of bladder cancer, and that is why it cannot be the first choice of treatment.

Therefore, patients with type 2 diabetes mellitus have to take the most suitable drugs for their health depending on many factors, including the patient’s age, the treatment goal, general health situation, how well the drug works, and how well it is tolerated [13].

While successful treatment and the proven advantages resulting from tight blood glucose regulation exist, the evidence suggests that the achievement of prescribed factor goals for patients with type 2 diabetes mellitus continues to be suboptimal [14].

Pharmacists represent the third largest health profession in the world [15].

Most pharmacists work in the community, with a smaller proportion in industry, academia, research, and in hospital pharmacies.

However, what constitutes a community-based pharmacy is much broader than the traditional retail setting.

A community pharmacist is defined according to the WHO as “the health professional most accessible to the public”. They supply medications following a prescription or when legally permitted, sell them without prescriptions [16].

Several studies have recognized the positive impression of pharmacists on preventative care, such as health screenings, immunizations, opioid management, smoking cessation efforts, and the management of chronic diseases, such as diabetes [17].

Therefore, special pharmaceutical care is needed for patients with type 2 diabetes mellitus regarding dose management, instruction on the correct use of medications, and pharmacokinetics. Through their experiences in pharmacotherapy and their community accessibility, pharmacists are capable of contributing to the enhancement of diabetes treatment with specialists through various techniques [18].

The public health role of the pharmacist is yet to be well-defined and is mostly documented and sufficiently promoted by public health agencies, pharmacy educators, or other health care professionals. Through their community accessibility, pharmacists have the highest level of patient contact, as no appointments are required to see them. They work in a variety of public settings. This convenience creates a large window of opportunity to provide public health services, filling a void related to access to care and prevention [19].

As the scope of pharmacy services shifts towards a greater emphasis on direct patient care, pharmacy diabetes services are one of the most important multidisciplinary approaches that resulted in a significant enhancement in clinical outcomes for diabetic patients [20]. For example, community pharmacists offer their patients diabetes-testing supplies (DTS) to aid patients with diabetes [21].

In addition, given the number of people living with prediabetes, community pharmacists are in an ideal position to increase recruitment and enrollment in the National Diabetes Prevention (DPP) Lifestyle Change Program, particularly for underserved populations [22]. These services enable community pharmacists to become highly trained and accessible healthcare professionals that can provide a clinically effective professional service for people with type 2 diabetes mellitus and support public health goals by increasing the awareness of the disease.

This systematic review will address the following research question: what is the impact of pharmacists’ interventions on type 2 diabetes patients’ therapeutic adherence and glycated hemoglobin HbA1c levels in primary care settings?

This finding will provide a general indication of the types of interventions that have been realized in the community pharmacy setting and their effectiveness.

Consequently, the main objective of this systematic review is to estimate the association between interventions led by community pharmacists and the mean change in patient adherence levels. The secondary objective is to evaluate the association between these interventions and the mean change in HbA1c levels.

**Figure 1:**
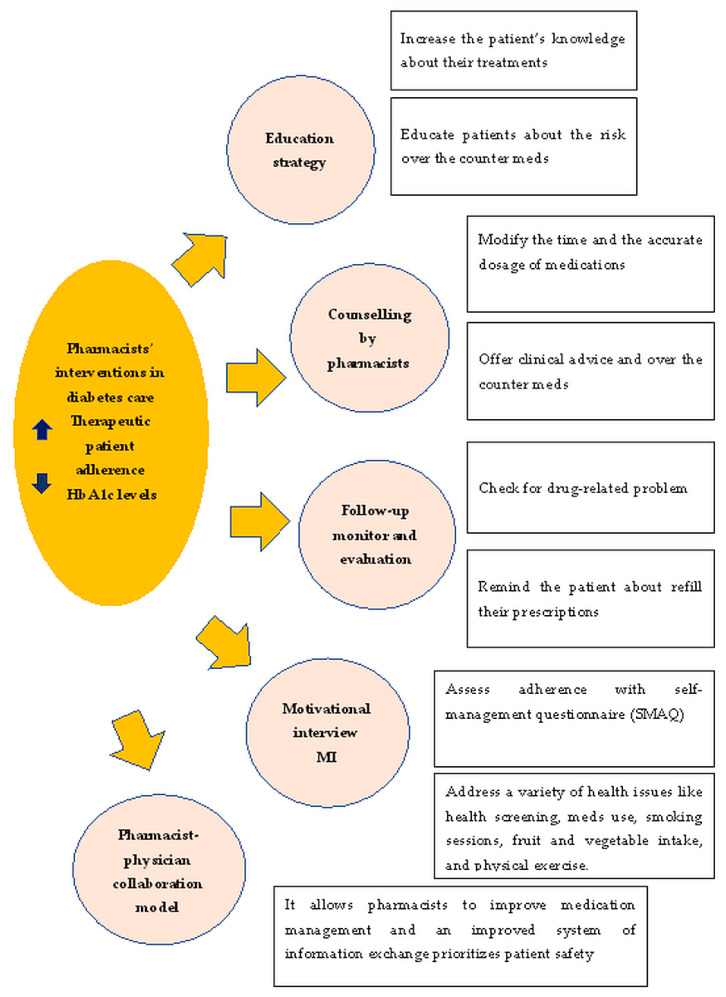
The most common strategies of pharmacists’ interventions in diabetes primary care. Abbreviations: MI, motivational interview; SMAQ, self management assessment questionnaire.

Based on our review, Figure 1 represents the most common pharmacist interventions in diabetes care via different strategies.

## 2. Materials and Methods

The systematic review protocol was developed based on the preferred reporting items for systematic review and meta-analysis (PRISMA, 2020) guidance [23].

### 2.1. Data Source

A cumulative search for studies was carried out in the following databases:

PubMed/Medline, Web of Science, and CINAHL between 2010 and 2020. The keywords “type 2 diabetes”, “therapeutic adherence” and “pharmacist” were used, combined with the Boolean operator “AND”. The references from all the electronic searches were downloaded into EndNote X7 reference manager. The detailed search strategy is given in Appendix A.

### 2.2. Eligibility Criteria

The following criteria were set for articles to be eligible for inclusion in this systematic review: original research studies published in peer-reviewed journals, while review articles, conference papers, editorials, and commentaries were not included.

The inclusion criteria were based on participants, intervention, comparison, outcome measures and study design, as presented below.

#### 2.2.1. Types of Participants

Participants with type 2 diabetes mellitus only were included in the study.

There were no restrictions imposed based on race, or sex, but we excluded pregnant women and children. With regard to age, we only included adults >18 years old.

#### 2.2.2. Types of Intervention and Comparator

The studies discussing strategies lead by community pharmacists in the primary care setting for type 2 diabetes mellitus patients were compared with usual pharmaceutical care. To be included, each study should have reported the mean change in the patient’s adherence level and the mean HbA1c levels at the baseline and at the end of the study.

#### 2.2.3. Types of Outcome Measures

Quantitative reporting behavior and clinical outcome introduced the following behavior outcome: patient adherence to the prescribed medications.

The clinical outcome was as follows: the effectiveness of pharmacist intervention on glycated hemoglobin (HbA1c) levels only.

#### 2.2.4. Types of Study Design

Randomized controlled trials, non-randomized control trails, quasi-controlled trials, cluster-controlled trials, before-and-after studies, case–control, retrospective and prospective cohort studies were included in this review. First, studies were selected depending on the title and abstract. We included only peer-reviewed studies that presented existing models of pharmaceutical interventions in diabetes primary care. Thestudies without control groups were excluded.

### 2.3. Exclusion Criteria

Reviews or case reports, full text unavailability, studies without control groups, studies with no results provided or studies not in the English language were excluded. Studies in which intervention was not delivered by community pharmacists were also excluded. In addition, pregnant women and children were excluded. Finally, studies without relevant outcomes, e.g., medication adherence measured using a non-validated tool, were excluded.

### 2.4. Study Selection

We used a PRISMA 2020 flow diagram to extract the most relevant data essential for synthesizing the results. First, all the results obtained from all the databases were exported to the EndNote X7 reference manager, and the duplicated studies were removed. Two researchers performed the data synthesis. The titles and abstracts were screened for eligibility by one author and around 5% were independently screened by another author.

The studies were divided into the following two categories: ‘definitely include’ and ‘definitely exclude’, followed by full text retrieval analysis. The full text articles in the ‘definitely include’ category were obtained. The results were assessed independently for final eligibility using the inclusion/exclusion criteria, and the reasons for exclusion were recorded.

To obtain the studies that were missed by the electronic literature searches, we also manually searched journals. The original authors were not contacted for further information.

### 2.5. Data Extraction

Data from the included studies were extracted to a table via Excel Software. (Microsoft Excel 2010, Microsoft Corporation, Washington, USA) The extracted data comprised title of articles, study design, duration, pharmacists’ interventions, methods in measuring medication adherence, outcomes (patient adherence, mean HbA1c levels) and reported results.

The extraction form was completed by S.A. and reviewed by R.Z.

### 2.6. Quality Assessment and Data Analysis

Based on our inclusion criteria, a risk of bias assessment accompanied each included study, using Cochrane guidelines [24].

The risks were identified as ‘high risk’, ‘low risk’ and ‘unclear risk’. Assortment, performance, detection, abrasion and reporting biases were tested.

The studies at “high risk” were discussed amongst the authors to certify suitability in the final review. Likewise, the studies were paralleled based on their study design, results and the interventions assumed.

The use of meta-analysis for assembling the results was unsuitable as the pharmacists’ interventions and methods of measuring adherence were different amongst the studies.

### 2.7. Ethics and Dissemination

No ethical approval was necessary to obtain the data because this systematic review did not involve patient personal data. The results will be disseminated by the publication of the manuscript in a peer-reviewed journal.

## 3. Results

The literature search identified 8362 papers, among which (*n* = 8069) from Web of Science, (*n* = 97) from CINAHL, and (*n* = 196) from PubMed/Midline.

A review of these titles and abstracts retained (*n* = 103) the manuscripts for detailed analysis. After ensuring the inclusion criteria, 21 papers remained in this systematic review.

We created groups by EndNote reference manager to help with handling the included and excluded studies. A group of the included abstracts was made, covering studies in the English language, and the remaining studies were excluded for not meeting the inclusion criteria. Subsequently, from the included abstracts group, we generated a group of the included full texts, containing (*n* = 21) studies associated with the topic and outcomes.

**Figure 2:**
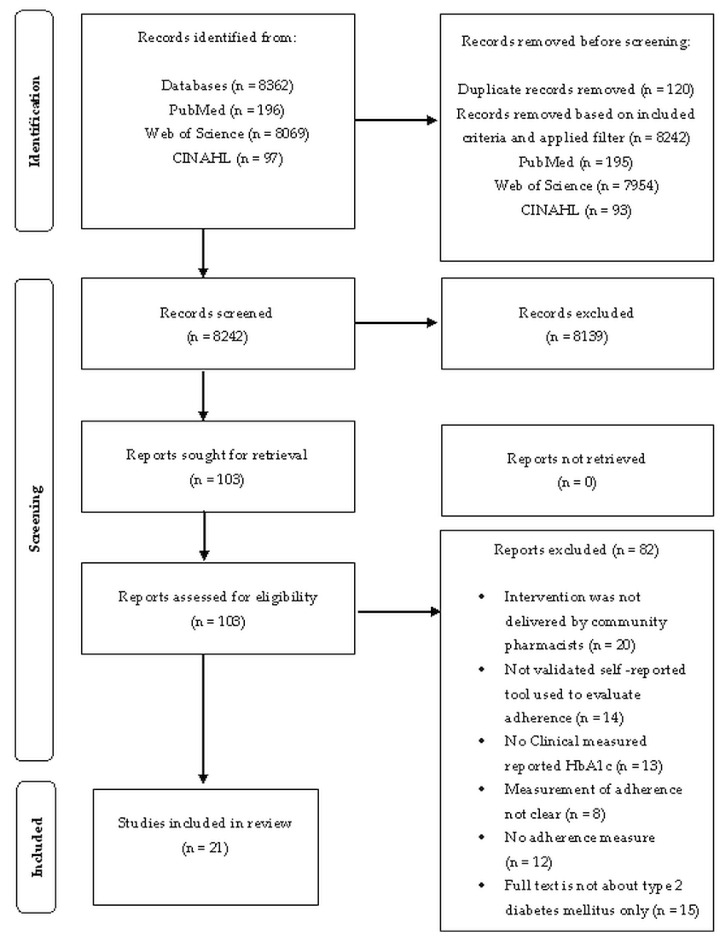
PRISMA-2020 flow diagram, which shows the relevant articles included in the study.

Some studies were excluded (*n* = 82). The foremost reasons for exclusion were interventions that did not correspond to the inclusion criteria (e.g., clinical pharmacist interventions, secondary or tertiary care); no data on HbA1c in each group and measurements of patient adherence that were not clear. The details are presented in Figure 2.

The PRISMA flow chart in Figure 2 shows the results for screening, the selection of papers, and reasons for exclusion.

### 3.1. Study Characteristics

The 21 studies involved in this review were conducted in different countries all over the world. Two studies were conducted in Europe (United Kingdom and France) [25,26], three in the USA [27,28,29], one in Australia [30], two in Brazil [31,32], one in New Zealand [33], twelve studies in the Middle East and Asia (Malaysia, Iran, Pakistan, India, Ethiopia, South Thailand, Cyprus and Jordan) [34,35,36,37,38,39,40,41,42,43,44,45], respectively.

Most of the studies were randomized controlled trails. Each study involved in the review assessed patient adherence and HbA1c levels as outcomes. Additionally, the majority of the studies took a place in primary diabetic care clinics/centers.

Table 1 summarizes the characteristics of the studies comprised in our review.

### 3.2. Risk of Bias Assessment

The value of all the qualified studies was tested by the Cochrane quality tool against each of the following criteria: random sequence generation, allocation concealment, blinding of participants and personnel, blinding of outcome assessment for each outcome, incomplete outcome data and selective reporting.

The blinding of participants and personnel was a domain that was frequently identified as being at ‘high risk’ of bias in the eligible studies.

Community pharmacists can carry out the intervention and evaluate the predominantly clinical outcomes, such as measurement of blood glucose level using the test kits that exist in the pharmacy, or offer a screening service to test whether you are diabetic or at risk of developing type 2 diabetes.

Furthermore, they can also administer questionnaires to patients for self-reported adherence.

Due to the nature of the interventions conducted by community pharmacists, patients were often unblended and mindful of their allocation into the intervention or control groups.

Meanwhile, all the studies provided the patients with information before contributing; therefore, the patients could easily define this allocation and would have known how their adherence and/or clinical outcome is going to be assessed.

### 3.3. Qualitative Synthesis Outcomes

The studies included in this review assessed the impact of community pharmacist-led interventions on patients’ medication adherence and glycated hemoglobin HbA1c levels. The outcomes described in these studies included behavioral and clinical outcomes. Table 1 demonstrates the study outcomes and their statistical significance.

### 3.4. Pharmacists’ Interventions

#### 3.4.1. Education

This strategy focuses on increasing diabetic patients’ knowledge about their treatment, drug side effects (hypoglycemia), how to take their treatment in accurate dosages, in addition to addressing a patient’s beliefs concerning pros and cons of therapy.

Educational methods have been known as the foremost approach by pharmacists to address a patient’s needs. This approach was able to elicit several patients’ recommendations to enhance their adherence to diabetes medicines.

Several studies showed that the education of a patient by a pharmacist brings about a significant change in therapeutic adherence status [39,40].

#### 3.4.2. Counseling by Pharmacist

Pharmacy counseling services are essential to a patient’s understanding of their medications. In the literature, more than one study showed that pharmacists have a very important positive role in counseling type 2 diabetes patients [31]. For example, a face-to-face counseling session provided by a pharmacist and regarding knowledge on diabetes, self-monitoring of blood glucose, and a regular checkup showed a positive influence on therapeutic adherence, and resulted in a measurable decline in HbA1c levels. In addition, the pharmacist also gave counseling regarding non-pharmacological management strategies, such as diet control, exercise therapy, and early identification of symptoms of hypoglycemia. In this way, all the patients were educated regarding antidiabetic medications and their indications.

Moreover, in the counseling session, the pharmacist also attempted to improve medication adherence in patients by tailoring the medication administration time and dosage according to the patient’s needs, as shown by as shown by Narayana Goruntla et al. [41].

Similarly, Samtia, Rasool et al. indicated that pharmacist counseling could play a crucial role in improving glycemic control for diabetic patients [38].

#### 3.4.3. Pharmacist—Physician Collaborative Care Model

Improved communication specifically between the pharmacist and physician allows pharmacists to improve medication management. A study conducted by Mouhtadi et al. showed that the collaborative care model between the physician and the pharmacist was successful in reducing FBG and improving patient satisfaction [46].

Aguiar et al. found that the collaborative care model is feasible and more effective than the usual care in the reduction in HbA1c levels in patients with uncontrolled type 2 diabetes mellitus [31].

#### 3.4.4. Family Support Led by Pharmacist

This strategy highlighted family-involvement intervention, which is very helpful in diabetes management. This intervention was observed in a RCT study that resulted in a greater reduction in glycosylated hemoglobin HbA1c, and a significant improvement in medication adherence [43].

#### 3.4.5. Motivational Interview (MI), and Telephone-Led Intervention by Pharmacist

MI is the most widely recognized method for improving long-term medication adherence. This intervention was observed in a study that showed a positive significant effect on medication adherence, but did not significantly improve HbA1c levels [27].

On the other hand, telephone-led intervention by a pharmacist was observed in three randomized control trails studies and of these, two studies resulted in a positive effect on improving patient’s adherence, but did not significantly improve HbA1c levels [25].

However, the third RCT study led by trained pharmacists resulted in a positive significant change in both the HbA1c levels and patient’s adherence [36].

#### 3.4.6. Simplicity of Complex Medication Regimes

The prevalence of a high medication-regimen complexity index is high among patients with chronic illness, particularly type 2 diabetes mellitus. The most affordable cause for non-adherence medication therapy was using diabetes-specific medication regimen complexity.

For this reason, physicians and pharmacists have to increase adherence to precise medications and improve glycemic control through the simplification of complex medication regimens specifically for patients with diabetes [42].

#### 3.4.7. Pharmaceutical Care Intervention

This strategy emphasizes the role of trained community pharmacists who carry out interventions by reviewing the patient’s medication regimen and providing customized tutoring and training on the proper methods to take their medications.

Many experiences had a positive impact, increasing patients’ adherence and refining glycemic control levels [32,34,45].

Indeed, pharmacist-led care programs have been shown to help patients with diabetes succeed in achieving treatment goals and improving outcomes. In this review, five studies displayed a favorable significant impact of pharmacist-led diabetes programs and these programs were associated with improved patient adherence to medication, and reduction in HbA1c [35,37,44].

#### 3.4.8. Self-Management Support Intervention Led by Community Pharmacists

This strategy highlights the efficacy of programs that are offered by community pharmacists to help the patient effectively self-manage aspects of their diabetes, including motivation to lose weight, knowledge of correct medication use, diet, and regular exercise.

This intervention was observed in an RCT study that resulted in a greater reduction in glycosylated hemoglobin HbA1c and a significant improvement in patient adherence [30]. Table 2 presented the effect of community pharmacists’ interventions on patient adherence and HbA1c levels.

## 4. Discussion

This systematic review aimed to evaluate community pharmacists’ strategies to enhance therapeutic adherence and glycemic level by measuring HbA1c levels for type 2 diabetes mellitus patients. We can declare that many pharmaceutical care approaches seem efficacious and beneficial to increase patients’ adherence, control glycemic levels, and improve knowledge about diabetes. Twenty-one studies were recognized in this review by multiple pharmacists’ interventions.

Approximately 90% of the included studies in this review reported a significant impact of the pharmacists’ interventions on patients’ therapeutic adherence. Some studies showed a positive impact of the pharmacists in enhancing therapeutic adherence and glycemic control in the intervention group.

These studies included several interventions, such as face-to-face interviewing [27], pharmaceutical consultation [38], community pharmacists’ services [30], educational sessions [40], diabetes programs led by pharmacists [35], and remote telephone support [36].

It is important to highpoint that we only included studies that evaluated both outcome measures for patients with type 2 diabetes mellitus who are taking oral antidiabetics treatment. The studies of patients who are taking insulin treatment were removed from this review.

In all the surveys and questionnaires, the pharmacist’s role was underlined in helping patients recognize their illnesses and their medications.

The total achievement level of improvement in glycemic levels due to pharmacist intervention was described as 68.75% for counseling interventions, 69% for education and 61% for medication management and telephone-based interventions, which reminded patients about refilling prescriptions on time.

Similar results were shown in a systematic review, stating that education, medication management and counseling by pharmacists were good, effective methods in improving HbA1c levels [47]. Indeed, a meta-analysis by Coutureau et al. also found that the interventions led by pharmacists in primary settings could improve glycemic levels for type 2 diabetes patients through education and management of their therapy [48].

Most of the studies in this systematic review that evaluated the impact of a community pharmacist-led intervention on patient medication adherence showed a statistically significant result, favoring the intervention group.

Similar results were shown in a review conducted by Meece et al. that highlighted the most effective strategies of pharmacists (e.g., education, counseling, motivational interview, and collaborative practice models) to improve patient adherence [49].

The most effective interventions to improve patient adherence and glycemic levels include a combination of components, such as education, counseling, face-to-face interviewing, simplification of treatment regimens, and follow-ups [35,37].

Most of the interventions assessed in this review focused on patient education and/or counseling, as well as other apparatuses; hence, they were multifaceted.

The majority of the reviewed studies were randomized controlled trials, and the randomization process was typically completed at the pharmacy level. This could be owing to authors attempting to minimize contamination by control patients receiving the intervention [25,26,29,30,31,34,35,36,37,41,43,44,45]

The majority of the included studies used the Morisky Medication Adherence Scale (MMAS), which has also been a basis for developing derived scales. Other studies either used the medication possession ratio (MPR), counting pills (CP) or the proportion of days covered (PDC) to measure the adherence level of patients. A study conducted by Lyons et al. used both objective measures self-report tools and the medication possession ratio [25].

The results of this systematic review show that pharmacist-led interventions in primary care settings can improve patient adherence and glycemic control for adults with type 2 diabetes mellitus.

To our knowledge, this is the first study that has given an overall view of the existing literature regarding community pharmacists’ interventions in primary diabetes care and has evaluated these interventions on both behavior and clinical outcomes.

The type of strategies followed by pharmacists, the tool used to measure patients’ adherence to treatments, and the results of clinical outcomes are considered effective elements in the results of this review.

A review conducted by Milosavljevic et al. found that the evidence supporting the positive impact of community pharmacy-based interventions on patients’ health outcomes and medication adherence is still limited, when compared to other health care settings. The review by Milosavljevic et al. was limited to studies published until October 2015 [50].

In contrast, our systematic review is different in terms of the inclusion criteria (i.e., types and number of studies included, types of participants, the outcomes reported, studies duration, and the published years). In addition, we have covered studies published worldwide. The emphasis is on the community pharmacy interventions’ efficacy on patient adherence and glycemic control levels (HbA1c) for type 2 diabetes patients only.

Concerning the barriers, Ilardo et al. found that lack of time, training, resources, unprofessional relationships, lack of collaboration models with other health professionals and deficiency of public awareness of the available services are the most significant barriers to the implementation of community pharmacist interventions [51].

The time intensive nature of pharmacists’ interventions makes the intervention strategy not appropriate to be implemented in community pharmacies, due to the high workload. For example, counseling by pharmacists for patients with chronic diseases is very complicated and takes more time, compared with patients who just want to know about the drug side effects. For this reason, setting appointments with pharmacists, writing a professional protocol including short message service reminders, and medication booklets might be great choices for the effective implementation of assured intervention.

Besemah et al. showed the effectiveness of the pharmacist primary health care intervention program for type 2 diabetes mellitus patients [52].

The focus was primarily on patients’ medications, medical conditions and the demonstration of effective techniques.

The results of this review indicate that the frequent follow-ups by pharmacists, educational sessions on drug therapy, monitoring of treatment, and reminding patients to refill their prescription on time were more likely to achieve positive satisfactory results and improve the quality of care.

The findings from this systematic review align with this concept, as the interventions led by community pharmacists positively influenced patient knowledge and satisfaction.

Either strategy used by pharmacists, including frequent contact with patients through phone calls or consultation, should be focused on in future studies.

Henceforth, a large effort is required for further examinations, which influence the perceived therapeutic adherence tool features related to diabetes mellitus treatment across diverse populations.

## 5. Limitation

The limitations are related to the time frame and the relatively low number of patients involved in each study. The authors of the cited articles explained that some only had a short period of intervention, which is not enough for monitoring long-term adherence. In addition, a small sample size is not adequate to give powerful results. Other studies justified these results by the tools they used for measuring medication adherence, which were considered not precise enough, such as self-report, and may give overestimations on medication adherence.

## 6. Conclusions

Pharmacists are in a unique position to play a very important role in accumulative patient therapeutic adherence. Likewise, as we tend to detect from this review, interventions led by community pharmacists have contributed to enhanced patient adherence, and reported better diabetes-related self-empowerment via enhanced medication knowledge and lifestyle modification due to better disease control.

For this reason, further research on pharmacists’ interventions must clarify the most effective elements, which are summarized by embracing therapeutic adherence approaches with usual daily activities and describing the type and reasons of the non-adherence problems section is mandatory. It means that future research should attempt to better understand the components that make the greatest contribution towards improving adherence and health outcomes for patients with different medical conditions. Based on the results, professional guidelines can be implemented in regular pharmacist services to improve the therapeutic outcomes of patients suffering from chronic diseases.

## Figures and Tables

**Table 1:** A summary of the characteristics of the studies comprised in our review.

Study Design	Study Duration	Sample Size	Pharmacist Intervention	Adherence Tool	Impact on Adherence	Reduction in HbA1c	Reference
Cluster-RCT	6-month follow-up	377 participants	Education	Medication possession ratio (MPR)	Did not significantly improve the already high adherence rates	Significant decreases in HbA1c (*p* < 0.01)	[26]
Open labelled interventional study	9-month follow-up	55 participants	Education	Validated questionnaire	Adherence significantly improved (*p* < 0.05)	Statistically significant reduction (*p* < 0.05)	[40]
Non-RCT	3-month follow-up	392 participants	Education	The 8-item Morisky Medication Adherence Scale	Adherence significantly improved (*p* < 0.05)	Statistically significant reduction (*p* < 0.05)	[39]
RCT	21-month follow-up	73 participants	Pharmacist-physician collaboration model	4-item Morisky–Green test	Adherence significantly improved (*p* < 0.001)	Greater reduction in HbA1c (*p* < 0.05)	[31]
Records analysis	48-month follow-up	115 participants	Follow-up by pharmacist	Medicines Use Review (MUR) Service	Adherence significantly improved (*p* < 0.05)	Greater Significant reduction (*p* < 0.05)	[33]
Case–control study	5-month follow-up	500 participants	Counseling by pharmacist	Self-reporting approach	Adherence significantly improved (*p* = 0.003)	Significantly improved HbA1c level (*p* < 0.001)	[38]
Prospective, open-labelled- RCT	3-month 6-month follow-up	330 participants	Counseling by pharmacist combined with message reminder	A pill count and visual analog scale (VAS) methods	Adherence significantly improved (*p* < 0.001)	Significantly reduced HbA1c (*p* < 0.01)	[41]
RCT	9-month follow-up	196 participants	Family support led by pharmacist	Self-reported Morisky Medication Adherence Scale (MMAS)	Adherence significantly improved (*p* < 0.05)	Significant reduction in HbA1c (*p* < 0.001)	[43]
A quasi-experimental intervention with a single-group design	6-month follow-up	28 participants	Motivational interview- strategy led by pharmacist	Self-reported diabetes medication adherence	Adherence significantly improved (*p* = 0.010)	Statistically significant reduction HbA1c (*p* = 0.090)	[27]
RCT	6-month follow-up	387 participants	Diabetes Medication Assistance Service (DMAS) Self-management support interventions (SMSI) delivered by the pharmacists	Brief Medication Questionnaire (BMQ)	Significantly improved (*p* < 0.05)	Significantly decreased (*p* < 0.001)	[30]
RCT	12-month follow-up	1400 participants	Impact of a telephone-based patient-centered intervention	Proportion of days covered (PDC)	Positive impact on adherence “slightly difference but not significant”	Not statistically significant in patients with poorly controlled diabetes	[29]
RCT	6-month follow-up	612 participants	Telephone consultations with a pharmacist	Self-reported adherence to medication Diagnostic Adherence to Medication Scale (DAMS) and medication possession ratio (MPR)	Adherence significantly improved (*p* = 0.010)	Statistically significant reduction (*p* = 0.061)	[25]
RCT	9-month follow-up	88 participants	A telephone-based intervention led by pharmacist	Morisky Medication Adherence questionnaire (8 items).	Adherence significantly improved (*p* < 0.05)	HbA1c significantly improved (*p* < 0.05)	[36]
A retrospective case–control study	12-month follow-up	100 participants	Pharmacist-directed medication therapy management (MTM)	Medication adherence was determined by anti-diabetes prescription refill	Adherence significantly improved (*p* < 0.001)	Significantly improved (*p* < 0.001)	[28]
Cross-sectional study	2-month follow-up	275 participants	Simplicity of complex medication regimes	Morisky Medication Adherence for Sub-Saharan counties (MMAS).	Low diabetes MRCI resulted in significantly increased adherence (*p* < 0.001)	High diabetes MRCI resulted in poor glycemic control	[42]
A prospective and experimental study	12-month follow-up	71 participants	Pharmaceutical care intervention (PC)	Morisky–Green test	Adherence significantly improved (*p* < 0.05)	A significant reduction in HbA1c (*p* < 0.05)	[32]
RCT	12-month follow-up	241 participants	Pharmaceutical care intervention (PC)	Malaysian Medication Adherence Scale (MMAS)	Adherence significantly improved (*p* = 0.007)	A significant reduction in HbA1c (*p* < 0.001)	[34]
RCT	6-month follow-up	106 participants	Pharmaceutical care intervention (PC)	Self-reported medication adherence (Morisky Scale)	Adherence significantly improved (*p* < 0.05)	HbA1c decreased significantly (*p* < 0.05)	[45]
RCT	5-month follow-up	85 participants	Pharmaceutical care intervention (PC)	Morisky Medication Adherence Scale (MMAS)	Adherence significantly improved (*p* < 0.05)	HbA1c significantly decreased (*p* = 0.0001)	[37]
RCT	6-month follow-up	73 participants	Pharmaceutical care intervention (PC)	Morisky scores and quality of life (QoL) scores	Adherence significantly increased (*p* = 0.02)	HbA1c reduced significantly from 9.66% to 8.47% (*p* = 0.001)	[35]
RCT	12-month follow-up	152 participants	Pharmaceutical care intervention (PC)	Self-reported medication adherence (Morisky–Green test)	Adherence significantly increased (*p* = 0.013)	A greater reduction in HbA1c (*p* < 0.001)	[44]

BMQ: Brief Medication Questionnaire; DSR: days supply remaining; MARS: Medication Adherence Report Scale; MEMS: Medication Event Monitoring Systems; MPR: medication possession ratio; MRA: medication refill adherence; PDC: proportion of days covered; RCT: randomized controlled trial; NRCT: non-randomized controlled trial.

**Table 2:** Summary of the effect of community pharmacists’ interventions on patient adherence and glycemic levels based on the literature review ^1^.

Type of Intervention	Impact of Pharmacist’s Intervention on Patient Adherence and Reduction in HbA1c Level	References
Education strategy by Pharmacists	Significant positive influence on Hba1c level, but no improvement in adherence level	[26]
	Significant positive influence on patient adherence and reduction in HbA1c levels.	[40]
Medicine Use Review Service (MUR)	Significant positive influence on medication adherence and reduction in HbA1c.	[33]
Counseling by pharmacist	Significant positive influence on patient adherence and reduction in HbA1c levels	[31,38,41]
Pharmacist–physician collaborative model	Significant positive influence on patient adherence and reduction in HbA1c levels	[31]
Family support led by pharmacists	Significant positive influence on patient adherence and reduction in HbA1c levels	[43]
Motivational interview strategy (telephone-led by pharmacist)	Significant positive influence on both adherence and HbA1c levels	[36]
	No significant difference on patient adherence, but significant improvement in HbA1c	[29]
	Positive impact on patient adherence, but no significant change in HbA1c levels	[25,27]
Medication therapy management program (MTM)	Significant positive influence on patient adherence and HbA1c levels	[28]
Simplicity of complex medication regimes	Significant positive influence on patient adherence and HbA1c levels	[42]
Pharmaceutical care intervention (PC)	Positive impact on patient adherence and reduction in HbA1c levels	[32,34,35,37,44,45]
Self-management support intervention led by community pharmacists	Significant positive influence on patient adherence and HbA1c levels	[30]

^1^ Sixteen studies showed a significant improvement in both adherence and HbA1c levels; three studies showed a significant improvement in adherence level, but no significant improvement in Hba1c level; two studies showed a significant improvement in HbA1c level, but no significant improvement in adherence level.

## Data Availability

Not applicable.

## Appendix A

### Appendix A.1. Search Strategy

PubMed/Medline: “type 2 diabetes mellitus” (MeSH Terms) OR (“type 2 diabetes” (All Fields) AND “mellitus” (All Fields)) OR “type 2 diabetes mellitus” (All Fields) AND “therapeutic adherence” (MeSH Terms) OR (“therapeutic” (All Fields) AND “adherence” (All Fields)) OR “therapeutic adherence” (All Fields)) AND (“pharmacists” (MeSH Terms) OR “pharmacists” (All Fields) OR “pharmacist” (All Fields)) 794 results.

Web of Science ((((ALL = (type 2 diabetes mellitus)) OR ALL = (type 2 diabetes)) AND ALL = (therapeutic adherence)) AND ALL = (pharmacists)) OR ALL = (pharmacist) 8070.

CINAHL (“type 2 diabetes mellitus or type 2 diabetes” TX ALL TEXT) AND (‘therapeutic adherence” TX ALL TEXT) AND (“pharmacists or pharmacist” TX ALL TEXT) 97.
